# Embedding atomic cobalt into graphene lattices to activate room-temperature ferromagnetism

**DOI:** 10.1038/s41467-021-22122-2

**Published:** 2021-03-25

**Authors:** Wei Hu, Chao Wang, Hao Tan, Hengli Duan, Guinan Li, Na Li, Qianqian Ji, Ying Lu, Yao Wang, Zhihu Sun, Fengchun Hu, Wensheng Yan

**Affiliations:** National Synchrotron Radiation Laboratory, University of Science and Technology of China, Hefei, P. R. China

**Keywords:** Ferromagnetism, Magnetic properties and materials, Two-dimensional materials

## Abstract

Graphene is extremely promising for next-generation spintronics applications; however, realizing graphene-based room-temperature magnets remains a great challenge. Here, we demonstrate that robust room-temperature ferromagnetism with *T*_C_ up to ∼400 K and saturation magnetization of 0.11 emu g^−1^ (300 K) can be achieved in graphene by embedding isolated Co atoms with the aid of coordinated N atoms. Extensive structural characterizations show that square-planar Co-N_4_ moieties were formed in the graphene lattices, where atomically dispersed Co atoms provide local magnetic moments. Detailed electronic structure calculations reveal that the hybridization between the *d* electrons of Co atoms and delocalized *p*_z_ electrons of N/C atoms enhances the conduction-electron mediated long-range magnetic coupling. This work provides an effective means to induce room-temperature ferromagnetism in graphene and may open possibilities for developing graphene-based spintronics devices.

## Abstract (web summary)

Graphene has shown incredible promise as ideal material for numerous fields; however its use in spintronics has been hampered by the lack of intrinsic magnetism. Here, Hu et al succeed in embedding Cobalt in the graphene lattice, creating robust room-temperature ferromagnetism.

## Introduction

Graphene is a two-dimensional (2D) layered material composed of a one-atom-thick hexagonal network of *sp*^2^-hybridized carbon^1,2^. It possesses many unique properties such as large specific surface area^3^, superior mechanical strength^4^, high intrinsic carrier mobility^5,6^, and thermal conductivity^7^. Great efforts have been devoted to promoting the applications of graphene in a diversity of fields including field-effect transistors^8^, chemical sensors^9^, fuel cells^10^, and so forth. Graphene is also envisaged to be a highly promising material for next-generation spintronic applications, owing to its extraordinary carrier mobility, long spin diffusion length, weak intrinsic spin–orbit coupling, and limited hyperfine interactions^11,12^. For practical applications in spintronic devices, the graphene-based material is required to be ferromagnetic above room temperature. Unfortunately, pristine graphene is intrinsically diamagnetic and lacks local magnetic moments^13,14^. Therefore, development of effective strategies to induce and manipulate the ferromagnetism of graphene has become a key issue to extend its applications in spintronics.

So far, various strategies have been attempted to realize ferromagnetic ordering in graphene, including utilization of atomic vacancies^15^, *sp*^3^ functionlization^16^, chemical doping^14,17^, surface adsorption^18^, and zigzag edges^19^. However, the magnetic moments induced by point defects or edges are relatively weak and unstable; and consequently the ferromagnetic ordering often collapses at rather low temperatures^14,16,17,20,21^. For instance, Grigorieva and colleagues^21^ produced high-density vacancy defects on graphene with ion beam radiation and realized a macroscopic magnetic moment of 0.001 $\mu _{\mathrm{B}}$/atom; but no ferromagnetic coupling was observed at temperatures as low as liquid helium (4.2 K). Tucek et al.^14^ found that S-doped graphene could only maintain ferromagnetic ordering at temperatures below 62 K, and Liu et al.^22^ demonstrated that only pure diamagnetism can be observed in N-doped graphene at 300 K. To improve the stability of ferromagnetic coupling, the common wisdom is to increase the content of defects so as to shorten the distance between weak local magnetic moments. However, an excessive content of defects would break the π-bonding networks and degrade the electric properties of graphene. In analogous 2D system of graphene like transition metal (TM) dichalcogenides, doping of magnetic TM (Fe, Co, and Ni) has been experimentally demonstrated to be an effective method for inducing stable room-temperature ferromagnetic ordering^23,24^. However, direct doping of TM atoms in graphene is impractical because graphene is not an ionic crystal like TM dichalcogenides and its honeycomb networks are maintained by strong C–C covalent bonds. This makes the energy barrier of embedding a single TM atom into pristine graphene lattices very steep. Indeed, previous attempts to produce TM-graphene compositions merely yielded TM atoms or clusters adsorbed on the surface of graphene^25,26^, where the magnetism is not intrinsic but mainly contributed by the metal clusters. Meanwhile the adsorbed TM atoms on graphene could be easily dissociated or coagulated at elevated temperatures. For example, graphene with Co atoms attached on the surface can maintain ferromagnetic ordering only below 10 K^26^. Hence, stable anchoring of magnetic TM atoms on graphene is important for the manufacture of graphene-based room-temperature ferromagnetic materials. Inspired by the metal-nitrogen (M-N) moiety strategies developed recently for atomically dispersed TM catalysts^27,28^, we speculate that doping TM with the aid of coordinated N might be a possible way to embed TM atoms into graphene lattices. As the Pauling electronegativity of N (3.0) is significantly higher than that of C (2.5), the TM atoms can be strongly bound by N atoms rather than by C atoms as observed in many TM-N-C systems^28,29^. Therefore, the TM-N_x_ moiety can be doped in graphene and act as the ferromagnetic centers. Previous density functional theory (DFT) simulations confirmed the structural stability of the heavy N-doped graphene at up to 1000 K^30^, further supporting the feasibility of TM-N_x_ moiety on building the graphene-based room-temperature ferromagnetic materials.

Herein, motivated by the above consideration, we propose a coordination atom assisted strategy by embedding magnetic TM into graphene lattices under the assistance of coordinated N atoms, where stable room-temperature ferromagnetism can be achieved in graphene. This strategy is exemplified by the single-metal-atomic Co-N_4_ moiety doped graphene, which was synthesized via an impregnation-pyrolysis method, and exhibits unprecedented ferromagnetic orderings with Curie temperature (*T*_C_) up to ∼400 K. A high saturation magnetization of 0.11 emu g^−1^ at 300 K (0.73 emu g^−1^ at 5 K) is obtained at the Co content of 0.4 at.%. Detailed X-ray absorption spectroscopy characterizations and multiple-scattering simulations confirm the embedding of Co atoms in the graphene lattices, via the formation of isolated Co-N_4_ units. Electronic structure calculations reveal that stable room-temperature ferromagnetic coupling is mediated by carriers and arises from the *d-p* orbital hybridization between the embedded Co and C/N atoms. Besides the ferromagnetic ordering, the Co-N_4_ doped graphene possesses unique electronic structure where only one spin channel crosses the Fermi level, which is beneficial for the generation of spin-polarized current in spintronics^31^. This deliberately designed strategy opens up an avenue for the development of graphene-based spintronic devices.

## Results

### Analysis of sample morphology and structure

**Fig. 1:**
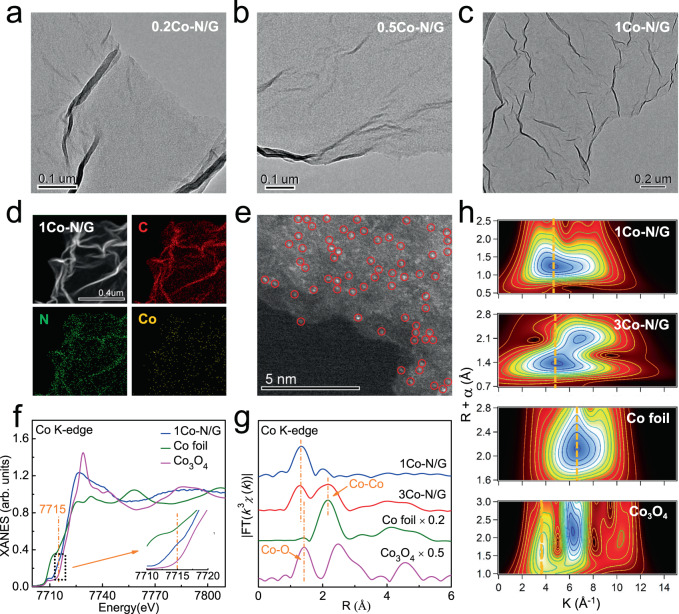
Atomic dispersion of Co atoms. TEM images of **a** 0.2Co-N/G, **b** 0.5Co-N/G, and **c** 1Co-N/G samples. **d** EDX elemental mapping and **e** HAADF-STEM image of 1Co-N/G; bright red circles represent isolated Co atoms. **f** Co K-edge XANES spectra for 1Co-N/G and reference samples. **g** FT *k*^3^-weighted Co K-edge EXAFS spectra and **h** WT analysis of EXAFS spectra for 1Co-N/G, 3Co-N/G, and reference samples.

The atomically dispersed Co embedded N-doped graphene (named 1Co-N/G) was prepared through a two-step synthesis strategy consisting of the impregnation and following pyrolysis procedures. For comparison, samples with other Co contents were prepared, denoted as 0.2Co-N/G, 0.5Co-N/G, and 3Co-N/G with 0.2×, 0.5×, and 3× Co precursors during preparation than that of 1Co-N/G sample (see experimental details in the Methods section). According to the X-ray photoelectron spectroscopy (XPS) analysis (Supplementary Table 1), the final Co contents in 0.2Co-N/G, 0.5Co-N/G, 1Co-N/G, and 3Co-N/G are 0.11, 0.22, 0.40, and 1.32 at.%, respectively. The transmission electron microscopy (TEM) images reveal transparent and corrugation sheets for all the samples (Fig. 1a–c and Supplementary Fig. 1a), typical morphology features for mono- and few-layered graphene-based materials. However, particles with an average diameter of ~20 nm are observed on the 3Co-N/G nanosheets (Supplementary Fig. 1b), indicating the formation of the second phase; meanwhile no particles were observed in the samples with lower Co contents. We used energy dispersive X-ray (EDX) spectroscopy and aberration corrected high-angle annular dark-field scanning transmission electron microscopy (HAADF-STEM) to further characterize the morphology of 1Co-N/G nanosheets. The EDX images (Fig. 1d) suggest the homogeneous distribution of C, N, and Co elements over the nanosheets. And in the HAADF-STEM image (Fig. 1e), a number of bright spots (some of them are highlighted by red circles for close observation) in single-atom size, corresponding to the isolated Co atoms, are well dispersed across the nanosheets. We studied the crystalline phase of the 1Co-N/G nanosheets using X-ray diffraction (XRD), observing a broad peak at about 26.3º (Supplementary Fig. 2) which can be indexed to the (002) plane of hexagonal graphite^32,33^. No Co-related second phases were detected. Furthermore, Co K-edge X-ray absorption near edge structure (XANES) spectra (Fig. 1f and Supplementary Fig. 3), Fourier-transformed (FT) *k*^3^-weighted Co K-edge extended X-ray absorption fine structure (EXAFS) spectra (Fig. 1g), and wavelet transform (WT) analysis (Fig. 1h) also show that Co exists as isolated atoms in graphene (see details in Supplementary Note 1). All the above TEM and XAFS results confirm that atomically dispersed Co in 1Co-N/G nanosheets have been successfully synthesized, without the formation of Co-related second phase when the Co loading is below 0.4 at.%. When Co content is beyond 0.4 at.%, part of the Co atoms are in the form of metallic Co nanoparticles (Fig. 1g, h and Supplementary Note 1).

**Fig. 2:**
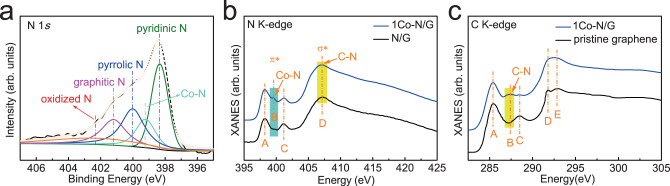
Bonding configuration of Co atoms. **a** N 1*s* XPS spectrum of 1Co-N/G nanosheets. **b** N K-edge XANES spectra of 1Co-N/G and N/G nanosheets. **c** C K-edge XANES spectra of 1Co-N/G nanosheets and pristine graphene.

**Fig. 3:**
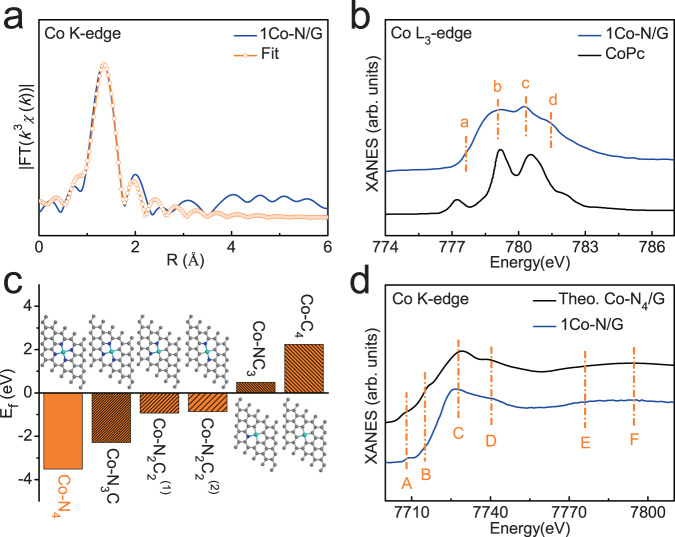
Coordination configuration around Co atoms. **a** Co K-edge EXAFS fitting curves of 1Co-N/G nanosheets in *R*-space. **b** Co L_3_-edge XANES spectra for 1Co-N/G and CoPc reference. **c** DFT calculated formation energies of various Co-N_4−*x*_C_*x*_ moieties in graphene lattices (Co-N_4−*x*_C_*x*_/G, *X* = 0, 1, 2, 3, and 4), together with the structural schematics. The cyan, blue, and black gray spheres represent Co, N, and C atoms, respectively. **d** Comparison of the experimental Co K-edge XANES spectrum of 1Co-N/G nanosheets and the simulated one based on a model of Co-N_4_/G.

In order to explore the bonding configuration of Co atoms in 1Co-N/G nanosheets, we performed XPS, and N and C K-edge XANES measurements. XPS survey spectrum (Supplementary Fig. 4) clearly shows the existence of C, N, and Co elements in 1Co-N/G nanosheets, while no other ferromagnetic impurities were detected. The corresponding fine-scan N 1*s* XPS spectrum of 1Co-N/G nanosheets shown in Fig. 2a, can be deconvoluted into five component peaks. Besides the N 1*s* signal from of pyridinic (398.3 eV), pyrrolic (400.0 eV), graphitic (401.2 eV), and oxidized N groups (402.3 eV), there is also a signal from Co-N bonds with the XPS peak at ~399.2 eV^32,34^. The formation of Co-N bonds was also confirmed by N K-edge XANES spectra (Fig. 2b), where an evident absorption peak B derived from Co-N bonding at ~399.5 eV can be observed, which is absent in the N/G nanosheets (see detailed N K-edge XANES analysis in Supplementary Note 2)^28,35^. Further, the C K-edge XANES features of 1Co-N/G nanosheets (Fig. 2c) are very similar to those of pristine graphene, indicating similar chemical environments of the carbon matrix, except for a feature at ~287.4 eV corresponding to the C-N bonding in the C K-edge XANES of the 1Co-N/G nanosheets (see detailed C K-edge XANES analysis in Supplementary Note 3)^36,37^. Taking these analysis together, we can conclude that there is a specific Co-N-C structural moiety in 1Co-N/G nanosheets. For quantitative analysis on the coordination configuration of Co atoms in 1Co-N/G nanosheets, a least-square EXAFS fitting was performed to extract the atomic structure parameters. Based on bonding configuration analysis, the best-fitting curves in *R*- and *q*-spaces for 1Co-N/G are shown in Fig. 3a and Supplementary Fig. 5 by using Co-N backscattering path. The average coordination number of N atoms is estimated to be 4 with an average scattering distances of 1.95 Å (see details in Supplementary Table 2). It is worthy to note that, as displayed in Fig. 1f, one pronounced pre-edge peak at ~7715 eV can be observed in the Co K-edge XANES spectrum for 1Co-N/G nanosheets. This is a typical feature of the Co-N_4_ square-planar structure^28,38^, consistent with the EXAFS fitting results indicative of four N neighbors around a Co atom. The square-planar Co-N_4_ configuration in 1Co-N/G could be further supported by the Co L_3_-edge XANES spectrum as shown in Fig. 3b, exhibits similar fine structure features (a, b, c, and d) to those of cobaltous phthalocyaninate (CoPc), but significantly different from those of Co metal and oxides as shown in Supplementary Fig. 6. This indicates that Co atoms are in a similar square-planar crystal field; the difference of crystal field splitting energy (corresponding to the distances between subpeaks) comes from the difference of ligands^39,40^. Furthermore, considering that the Co-C coordination cannot be unambiguously distinguished from the Co-N coordination by EXAFS analysis, DFT calculations were employed to check the stability of various CoN_4−*x*_C_*x*_ moieties in graphene lattices (CoN_4−*x*_C_*x*_/G, *X* = 0, 1, 2, 3, and 4) by comparing the formation energies (see calculation details in the Methods section). The calculation results and structural schematics are given in Fig. 3c, where the planar structure of each CoN_4−*x*_C_*x*_/G (*X* = 0, 1, 2, 3, and 4) moiety was ensured to be the converged configuration after structural optimization (see the details in Supplementary Figs. 7 and 8). It is obvious that the Co-N_4_ doped graphene (Co-N_4_/G) moiety is much more energetic favorable than other CoN_4−*x*_C_*x*_/G (*X* = 1, 2, 3, and 4) compounds. In addition, the simulated Co K-edge XANES spectrum based on the structure model of a square-planar Co-N_4_/G generated by DFT calculations is presented in Fig. 3d, with excellent agreement between the theoretical and experimental XANES spectra. All of the above experimental and theoretical results lead us to conclude that the square-planar Co-N_4_ moieties is the most possible structure for Co atoms in the 1Co-N/G nanosheets.

### Ferromagnetism activated by the Co-N_4_ moieties

**Fig. 4:**
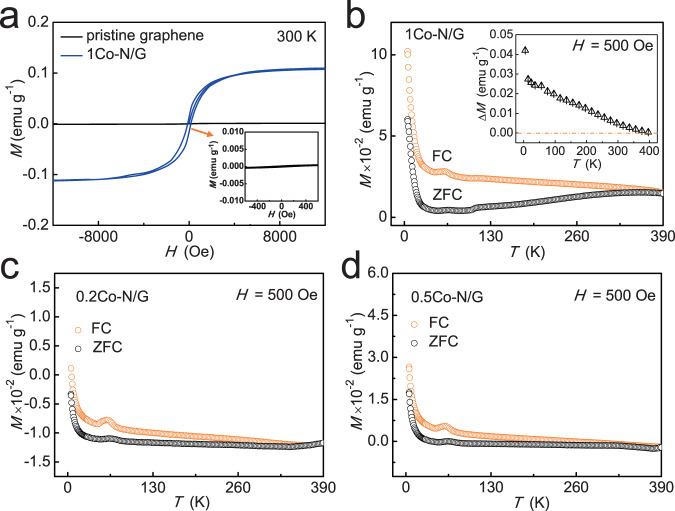
Magnetic properties. **a** Magnetization vs. magnetic field (*M*–*H*) curves for pristine graphene and 1Co-N/G nanosheets after background subtraction. The inset shows the *M*–*H* curves for pristine graphene in narrow scale for better perception. **b** Temperature dependence of FC and ZFC magnetization (*M*–*T*) curves for 1Co-N/G nanosheets. Inset: the magnetization difference (Δ*M*) between FC and ZFC curves (*M*_FC_–*M*_ZFC_) of 1Co-N/G nanosheets. **c**, **d** Temperature dependence of FC and ZFC magnetization (*M*–*T*) curves for 0.2Co-N/G (**c**) and 0.5Co-N/G (**d**) nanosheets.

Next, to clarify the effect of the Co-N_4_ moieties on the magnetic properties of graphene nanosheets, we measured the magnetization curves as a function of the applied magnetic field *H* (*M−H*) (Fig. 4a) at 300 K for pristine graphene and 1Co-N/G nanosheets. The well-defined hysteresis loop indicates the room-temperature ferromagnetic behavior of 1Co-N/G nanosheets with saturation magnetization and coercivity of ~0.11 emu g^−1^ and 142 Oe, respectively, different from the non-ferromagnetic properties of pristine graphene as shown in the inset illustration^21,22^. In addition, temperature-dependent magnetization curves (*M*−*T*) for 1Co-N/G nanosheets are given in Fig. 4b under field-cooling (FC) and zero-field-cooling (ZFC) modes at an applied field of 500 Oe; it is clear that the 1Co-N/G nanosheets have a *T*_C_ above room temperature (300 K). The magnetization difference (Δ*M*) between FC and ZFC curves (*M*_FC_−*M*_ZFC_) in the inset shows a decreasing trend but still perfectly positive in the whole temperature range (up to ~400 K). Hence, the possibility of spin glass effects and superparamagnetism can be excluded in the 1Co-N/G nanosheets^24,41^. The corresponding *M*−*T* curves for 0.2Co-N/G and 0.5Co-N/G under an applied field of 500 Oe are also depicted in Fig. 4c, d, and their *M*–*H* curves at 300 K are given in Supplementary Fig. 9, showing the diamagnetic background contributions in addition to the ferromagnetism, in analogy to previous reports^14,17^. Seen from Fig. 4b–d, it is obvious that all the three samples exhibit thermo-magnetic irreversibility (a bifurcation in the FC and ZFC curves) at temperatures above 300 K. And the cusp at about 65 K can be attributed to molecular oxygen on the surface^42–45^. Furthermore, in order to visually show the change of magnetism with Co contents in doped graphene nanosheets, we extracted magnetic signals from the *M*−*H* curves for all the samples and plotted them against the Co contents in Supplementary Fig. 10. Obviously, the saturation magnetization for as-synthesized samples is enhanced when the Co content increases from 0.2× to 1.0× Co. At the content of 3.0× Co, according to the above characterization analysis, the ferromagnetism is not intrinsic and partly comes from the contribution of Co-based nanoparicles or/and clusters. At the temperature of 5 K, the *M*−*H* curves for 1Co-N/G nanosheets exhibit similar ferromagnetism with higher saturation magnetization of ~0.73 emu g^−1^ (Supplementary Fig. 13). If there is ferromagnetism derived from Co metal clusters, by comparing the magnetic moment of 1Co-N/G samples measured at 5 K (∼0.4 μ_B_ Co^−1^) with that of Co metal (1.7 μ_B_ Co^−1^), we can estimate that ~24% of the total doped Co atoms should be in the form of clusters. Such a content of Co clusters should be easily detected by TEM and XANES^46^, which is however contrary to the experimental observations (Fig. 1c, f). Meanwhile, the *T*_C_ of 1Co-N/G at about 400 K is much lower than that of Co metal clusters. In addition, we treated intrinsic non-magnetic Al_2_O_3_ powder under the same experimental and magnetic characterization conditions as for the 1Co-N/G nanosheets. No ferromagnetism was observed, which helps ruling out the possibility of unintentional introduction of magnetic impurities during the experimental process. We can conclude that the ferromagnetism in the Co-N_4_ moiety doped graphene nanosheets is intrinsic.

### Origin of the ferromagnetism in CoN_4_-graphene system

**Fig. 5:**
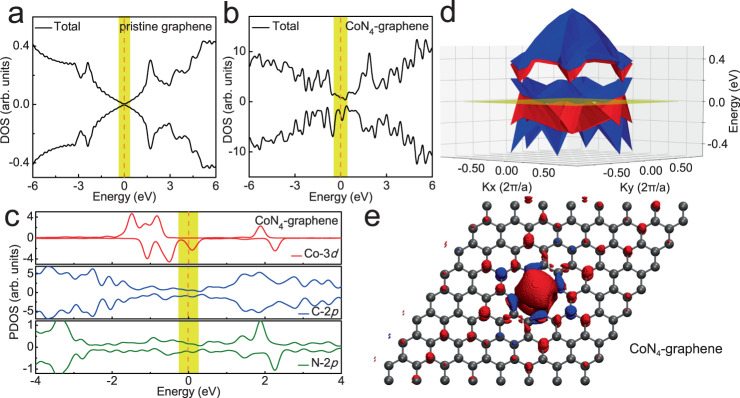
Electronic structure and origin of ferromagnetism. DFT calculated TDOS for **a** pristine graphene and **b** CoN_4_-graphene system. **c** DFT calculated PDOS for CoN_4_-graphene system. **d** 2D plot of the energy band structure for CoN_4_-graphene. The red and blue surfaces represent the bands with up and down spin directions, respectively. The transparent yellow plain corresponds to the Fermi level (*E* = 0 eV). **e** Spatial distribution of spin density (ρ↑−ρ↓) for a 8 × 8 × 1 hexagonal graphene supercell with one doped square-planar Co-N_4_ structure. Red and blue isosurfaces represent positive and negative spin densities, respectively.

To obtain an in-depth understanding on the origin of the room-temperature ferromagnetic coupling in CoN_4_-graphene system, we performed spin-polarize DFT calculations (see calculation details in the section of Methods). The total densities of states (TDOS), projected densities of states (PDOS), energy band structure and spatial distribution of spin density are presented in Fig. 5a–e, respectively. For reference, Fig. 5a shows the TDOS of pristine graphene, which has no spin polarization and is semi-metallic with Dirac cone at the Fermi level (corresponding to *E* = 0 eV in the DOS)^47,48^. In contrast, the TDOS and PDOS of CoN_4_-graphene system (Fig. 5b, c) exhibits a significantly enhanced and spin-polarized DOS around the Fermi level. According to the Stoner criterion^49,50^, a high DOS at the Fermi level ensures ferromagnetism at relatively high temperature. In order to show the band structure of CoN_4_-graphene more clearly, we made a stack diagram of DFT calculated total and projected DOSs for CoN_4_-graphene system (Supplementary Fig. 14). It is obvious that the system exhibits a metallic character with non-zero DOS at the Fermi level. In addition, we calculated the 2D plot of the energy band structure for CoN_4_-graphene as shown in Fig. 5d, where the Fermi level crosses some energy bands and no significant bandgap can be observed, consistent with the DOS (Fig. 5b, c and Supplementary Fig. 14) and previous theoretical calculations^51^. Further, the PDOS in Fig. 5c reveals the intense hybridization between the Co-3*d*, N-2*p*, and C-2*p* electronic bands. The spatial distribution of spin density (Fig. 5e) also indicates that the spin-polarized electrons in the graphene matrix are mainly delocalized *p*_z_ electrons. Bipartite arrangements of spin polarization can also be observed in the spatial distribution of spin density; the N atoms dictate the arrangement of spin polarizations in its own sublattices. Hence the magnetic states between two Co atoms should depend on their relative positions^52^, which is proved by the calculated relative energies of ferromagnetic (FM) and antiferromagnetic (AFM) states of different bi-Co-N_4_ configurations (Supplementary Fig. 15). The coexistence of FM and AFM states agrees with the fact that the measured magnetic moment is smaller than the predicted value (1.0 μ_B_/Co)^51,53^. Due to the intense hybridization between Co *d* and delocalized N/C *p*_z_ orbits, the long-range magnetic coupling between Co atoms can be attributed to an indirect conduction-electron mediated Ruderman–Kittel–Kasuya–Yosida (RKKY)-like interaction^54,55^. Meanwhile, we have also carefully considered other magnetic coupling frameworks including successive spin polarization-based ferromagnetic coupling (SSP-FMC)^56,57^ and bound magnetic polaron (BMP)^58^ that have successfully interpreted the origins of ferromagnetism in many TM-doped system. However, due to the metallic nature, enhanced DOS at Fermi level and spin-polarized *π* electrons for our CoN_4_-graphene system, the conduction-electron mediated ferromagnetism is a more reasonable and straightforward explanation for the main origin of ferromagnetism in CoN_4_-graphene nanosheets. Since the hybridization between the Co-3*d*, N-2*p*, and C-2*p* electronic bands is essential for the development of the ferromagnetic coupling in CoN_4_-graphene nanosheets, the strong room-temperature ferromagnetism originates from the chemical bonding that embeds Co atoms into the graphene lattice. Hence the DFT simulations lead us to conclude that the Co-N_4_ moieties are responsible for the observed room-temperature ferromagnetism in the as-synthesized CoN_4_-graphene nanosheets.

Finally, we summarize the important role of co-doped N atoms in the establishment of graphene magnetism: (1) N atoms serve as a stable anchoring site for Co due to the higher Pauling electronegativity than that of C atoms, which avoids the loss of ferromagnetic Co atoms from graphene lattice. (2) Compared with C atoms, N possess one additional *p* electron, making them an n-type dopant in graphene, which can effectively improve the electric conductivity of graphene and enhance the DOS around the Fermi level to meet the Stoner criterion. (3) In the context of the bipartite nature of graphene, the N atoms induce a bipartite arrangement of magnetic moments of the sublattices where they are. Moreover, it is worth noting that the CoN_4_-graphene nanosheets have greatly improved performance of spin-polarized current because only one spin channel crosses the Fermi level^31^.

## Discussion

In summary, we have explored the possibility of achieving stable room-temperature ferromagnetic ordering in graphene by embedding single magnetic TM atoms in the lattice via the strong chemical bonds in the TM-N_x_ moieties. A simple and controllable impregnation-pyrolysis process was employed to synthesize the Co-N-graphene system. Comprehensive structural characterizations confirm the existence of square-planar Co-N_4_ moieties in the graphene lattices at the content of 0.4 at.% (for 1Co-N/G nanosheets), which displays unprecedented stable ferromagnetic ordering with *T*_C_ up to ∼400 K and saturation magnetization of 0.11 emu g^−1^ (300 K). No Co-related second phase that may induce extrinsic ferromagnetism is observed. On the one hand, Co-N_4_ moieties avoid the loss of Co atoms from the graphene lattices, thus providing a strong local magnetic moment; on the other hand, DFT calculation reveals that the long-range ferromagnetic coupling originates from the hybridization between the Co-3*d* and C/N-2*p* orbitals through a carrier-mediated magnetic interaction. The stable room-temperature graphene magnets developed in this work will pave the way towards practical graphene-based spintronic applications.

## Methods

### Synthesis of 1Co-N/G and N/G nanosheets

1Co-N/G nanosheets were synthesized through a two-step impregnation-pyrolysis procedure. In the typical procedure, graphene oxide (GO) was firstly prepared by oxidation of powdered flake graphite following a modified Hummers’ method^59^. Then, 50 μl of CoCl_2_·6H_2_O solution (3 mg ml^–1^) and 15 μl of H_2_O_2_ solution (30%) were added dropwise into 10 ml of GO suspension (2 mg ml^–1^) and the mixture was sonicated for 1 h. The obtained suspension was transferred into a stainless steel autoclave and maintained at 180 °C for 6 h, forming a hydrogel. After freeze drying, the Co^2+^-containing gel was heated in the center of a quartz tube furnace at 900 °C for 1 h under gas flows of 100 sccm Ar and 50 sccm NH_3_ to obtain 1Co-N/G. For comparison, the N/G nanosheets were prepared with the same procedure with no addition of CoCl_2_·6H_2_O.

### Synthesis of 0.2Co-N/G, 0.5Co-N/G, and 3Co-N/G nanosheets

0.2Co-N/G, 0.5Co-N/G, and 3Co-N/G nanosheets were synthesized with a similar procedure to 1Co-N/G except for 0.2×, 0.5×, and 3× Co precursors during preparation, respectively.

### DFT calculation details

The spin-polarized DFT calculations were performed with Quantum Espresso software package^60^ and some calculation results were visualized with VMD software^61^. The electron exchange-correlation was processed within the framework of generalized gradient approximation (GGA) in the Perdew–Burke–Ernzerhof (PBE) parametrization. The electron-ion interaction was described using the projected augmented wave (PAW) method. The kinetic energy cutoffs of the plane wave and electron density were 80 and 500 Ry. The long-range van der Waals interaction was considered with the DFT-D3 scheme^62^. A 6 × 6 × 1 k-grid was used for sampling in the Brillouin zone. Energy convergence of ~1.0 × 10^−3^ meV/atom was ensured during the self-consistent field calculations. And the convergence criteria for the atomic forces was 0.05 eV/Å. Various typical planar and nonplanar configurations have been fully considered during the structure optimizations, as the examples shown in Supplementary Figs. 7 and 8.

The Co embedded nitrogen-doped graphene nanosheets were modeled based on an 8 × 8 × 1 hexagonal supercell of graphene. Six adjacent carbon atoms were replaced with Co-N_4_. A vacuum layer of 15 Å was placed along the z direction to prevent the interlayer interaction.

The formation energies of Co-N_*x*_C_4-*x*_ moieties in graphene were calculated according to the following equation,

1 $$E_f = E_{[{\mathrm{Co}} - {\mathrm{N}}_x{\mathrm{C}}_{4 - x}]} + \left( {2 + x} \right)\mu _{\mathrm{C}} - 64\mu _{\mathrm{C}} - x\mu _{\mathrm{N}} - E_{[{\mathrm{Co}}({\mathrm{g}})]}$$

where $E_{[{\mathrm{Co}} - {\mathrm{N}}_x{\mathrm{C}}_{4 - x}]}$ is the total energy of an 8 × 8 × 1 graphene supercell with a Co-N_*x*_C_4-*x*_ moiety; $\mu _{\mathrm{C}}$ and $\mu _{\mathrm{N}}$are the chemical potentials of C and N, defined as the total energy per atom in graphene and nitrogen molecules; ${\mathrm{E}}_{[{\mathrm{Co}}({\mathrm{g}})]}$ is the total energy of an isolated Co atoms.

### Characterization

TEM and EDX spectroscopy results were taken on a field emission transmission electron microscope (JEM-2100F) with 200 KV accelerating voltage. The HAADF-STEM images were captured on a JEM-ARM200F instrument at 200 KV. The XRD and XPS patterns were collected on a Philips X’Pert Pro Super diffractometer with Cu *K*α line (*λ* = 1.54178 Å) and an ESCALAB MKII equipped with Mg *K*α source (һν = 1253.6 eV), respectively. The N K-edge, C K-edge, and Mo L_3_-edge XANES were obtained at the BL12B beamline of National Synchrotron Radiation Laboratory (NSRL, China) under a total electron yield (TEY) mode with vacuum better than 5 × 10^−7^ Pa. The Co K-edge EXAFS spectra were obtained on the 1W1B beamline of Beijing Synchrotron Radiation Facility (BSRF, China) under fluorescence mode. The temperature- and magnetic field-dependent magnetization curves were measured on a superconducting quantum interference device (SQUID) magnetometer.

## Supplementary information

Supplementary Information

## Data Availability

The data that support the findings of this study are available from the corresponding author upon reasonable request.
