# Supplementary material for: Embedding atomic cobalt into graphene lattices to activate room-temperature ferromagnetism
Source: Nat Commun. 2021 Mar 25;12:1854. doi: 10.1038/s41467-021-22122-2 (PMC7994802; doi:10.1038/s41467-021-22122-2)
Supplement: Supplementary file 1 — Supplementary Information [file 41467_2021_22122_MOESM1_ESM.pdf]

Supplementary Information

Embedding atomic cobalt into graphene lattices to active room-temperature ferromagnetism

Wei Hu,[1] Chao Wang,[*,1] Hao Tan,[1] Hengli Duan,[1] Guinan Li,[1] Na Li,[1] Qianqian Ji,[1] Ying Lu,[1] Yao Wang,[1] Zhihu Sun,[*,1] Fengchun Hu,[1] and Wensheng Yan[*,1]

[1]National Synchrotron Radiation Laboratory, University of Science and Technology of China, Hefei 230029, P. R. China

Corresponding Authors

*E-mail: chaowng@ustc.edu.cn

*E-mail: zhsun@ustc.edu.cn

*E-mail: ywsh2000@ustc.edu.cn

Supplementary Notes

Supplementary Note 1. Detailed description of the existence of Co as isolated atoms in graphene by XANES spectra, FT k^3-weighted EXAFS spectra, and WT analysis

As shown in Fig. 1f and Supplementary Fig. 3, the Co K-edge XANES (Fig. 1f) and first derivative spectrum (Supplementary Fig. 3) for 1Co-N/G is significantly different from that of reference samples including Co foil and Co_3O_4, suggesting the positive valence state of Co atoms and excluding the possibility of metallic or/and oxide clusters of Co. The radial distribution functions of Co atoms abstracted from the Fourier-transformed (FT) k^3-weighted Co K-edge EXAFS spectra for 1Co-N/G, 3Co-N/G and reference samples are depicted in Fig. 1g. In accordance with the electron microscopy observation, no coordination shell of Co-O (~ 1.43 Å) or Co-Co (~ 2.16 Å) corresponding to Co-related second phases were detected for 1Co-N/G. In contrast, a coordination shell of Co-Co for 3Co-N/G indicates the existence of metallic Co nanoparticles, in agreement with the lattice spacing associated with hcp Co nanoparticles in the HRTEM images (Supplementary Fig. 1b). Further, in the wavelet transformed (WT) Co K-edge EXAFS spectra (Fig. 1h), the 1Co-N/G nanosheets shows only one intensity maximum at about 4.5 Å^{-1}. Meanwhile, intensity maximum at ~ 6.5 Å^{-1} corresponding to Co-Co coordiation[1,2] is absent in the 1Co-N/G, but can be observed in 3Co-N/G nanosheets.

Supplementary Note 2. Detailed N K-edge XANES analysis

The N K-edge XANES spectrum of 1Co-N/G nanosheets exhibits four distinct absorption peaks in Fig. 2b. The peaks A (at 398.2 eV), C (at 401.2 eV) and D (at 407.2 eV) can be assigned to the pyridinic N π^*, graphitic N π^* and C-N σ^* transition, respectively.[3,4] However, one pronounced absorption peak B associated with Co-N bonding at about 399.5 eV can be observed in the 1Co-N/G, while it is absent in the N/G nanosheets.[1,5]

Supplementary Note 3. Detailed C K-edge XANES analysis

The C K-edge XANES spectra of 1Co-N/G nanosheets exhibits five distinct absorption peaks in Fig. 2c. Among them, peak A at 285.4 eV is derived from graphitic C-C π* excitations; while peak D (at 291.8 eV) and E (at 292.8 eV) are derived from graphitic C-C σ* transitions.[6,7] The appearance of these peaks corresponds to carbon matrix.[7,8] In addition, peak C at about 288.5 eV can be ascribed to the O-C=O π*,[6] and its weak peak intensity indicates a low oxygen content in the 1Co-N/G nanosheets, consistent with the deconvoluted XPS results as shown in Fig. 2a. It is worthy to note that one noticeable peak B (287.4 eV) due to the π* resonance of C-N bond can be observed in the 1Co-N/G nanosheets while it is absent in the pristine graphene samples.[8,9,10]

Supplementary Figures

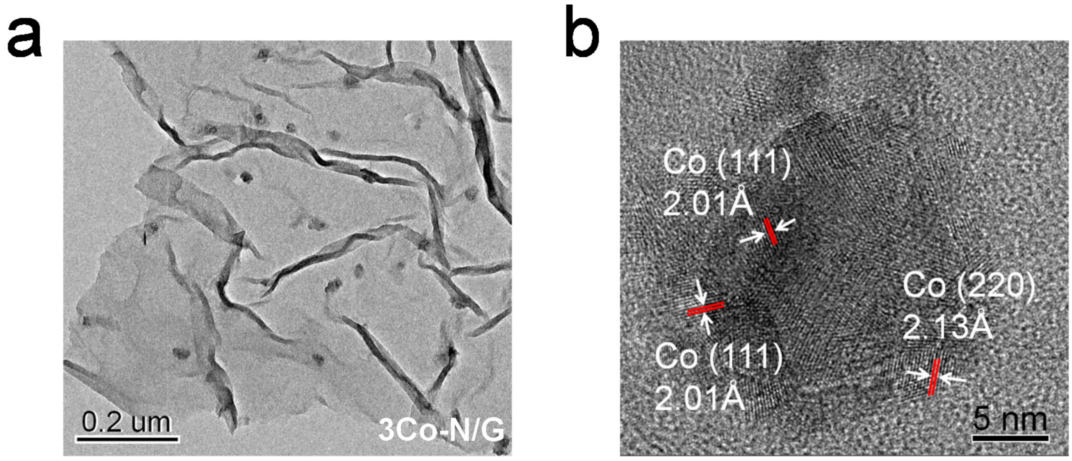

Supplementary Figure 1. Morphology characterization. a) TEM image of 3Co-N/G nanosheets. b) Corresponding HRTEM image with an expanded view on a single Co nanoparticle with diameter about 20nm in the 3Co-N/G nanosheets

The high resolution TEM (HRTEM) image in Supplementary Fig. 1b with an expanded view on the particles shows distinct lattice spacing at about 2.01 and 2.13 Å, corresponding to the (002) and (100) planes of hcp Co, respectively.

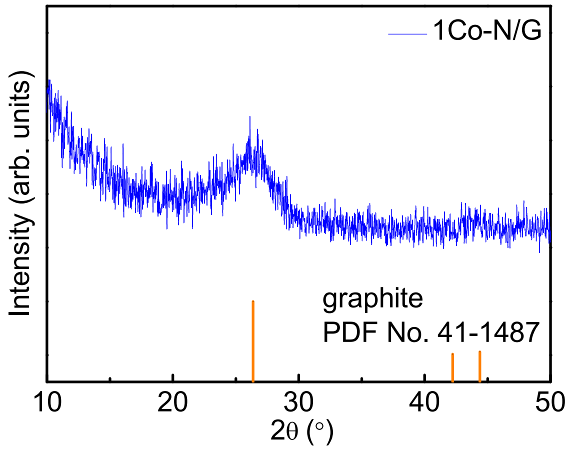

Supplementary Figure 2. XRD. XRD pattern of 1Co-N/G nanosheets.

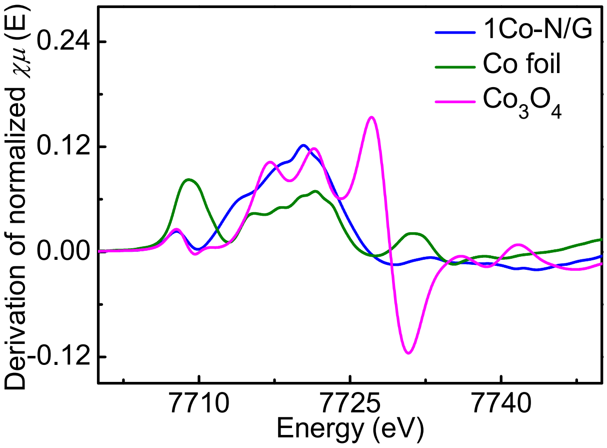

Supplementary Figure 3. Co K-edge XANES. First-derivative Co K-edge XANES curves for 1Co-N/G and reference samples including Co foil and Co_3O_4.

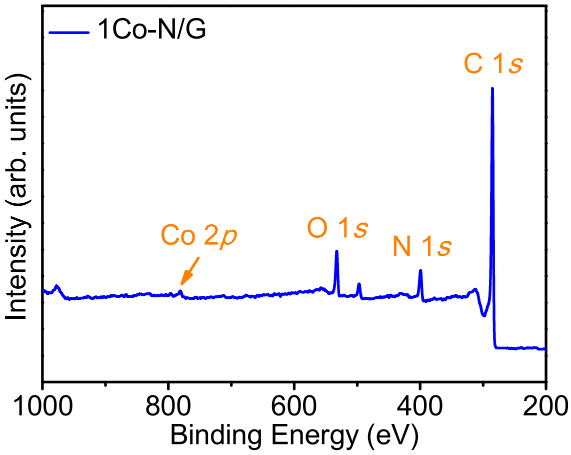

Supplementary Figure 4. XPS. XPS survey spectrum for 1Co-N/G nanosheets.

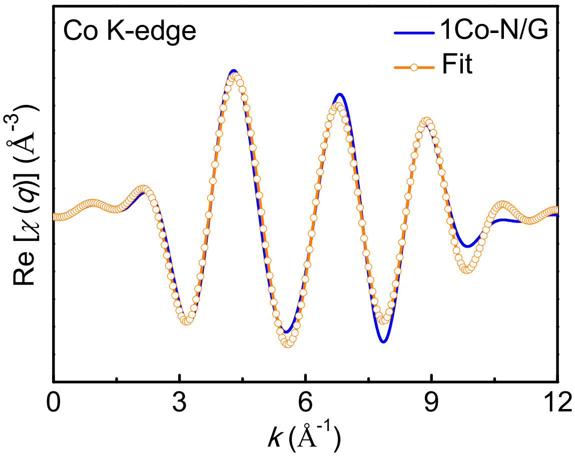

Supplementary Figure 5. EXAFS fitting. Co K-edge EXAFS fitting curves of 1Co-N/G nanosheets in *q*-space.

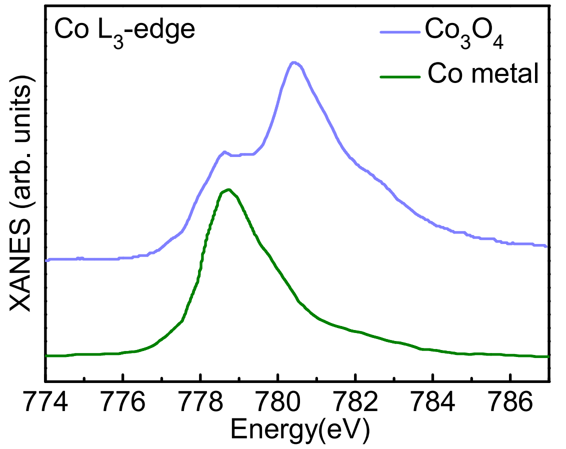

Supplementary Figure 6. Co L_3-edge XANES. Co L_3-edge XANES spectra for Co foil and Co_3O_4.

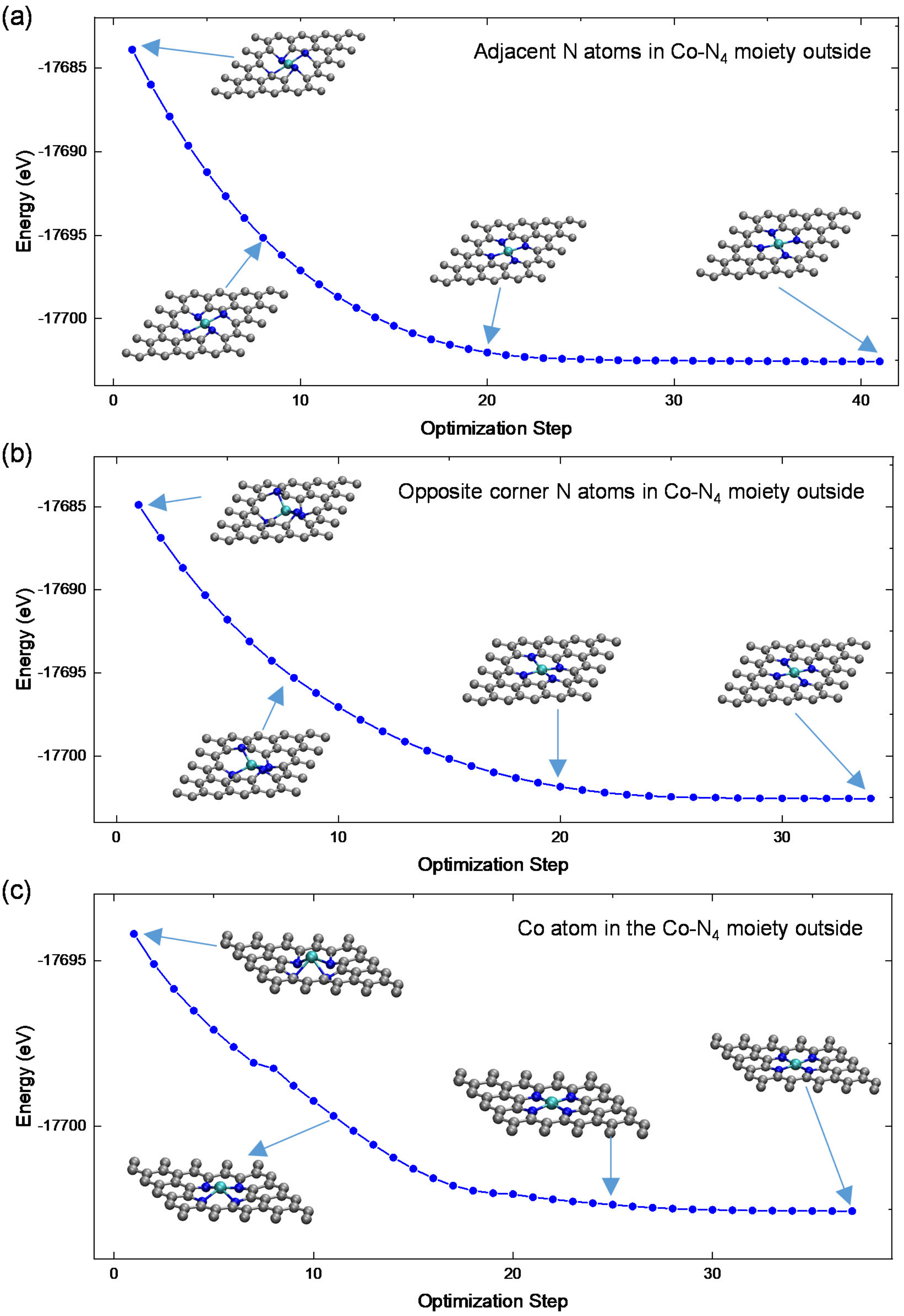

Supplementary Figure 7. Structural optimizations of Co-N_4. Structural optimizations with non-planar Co-N_4 moieties as initial structures.

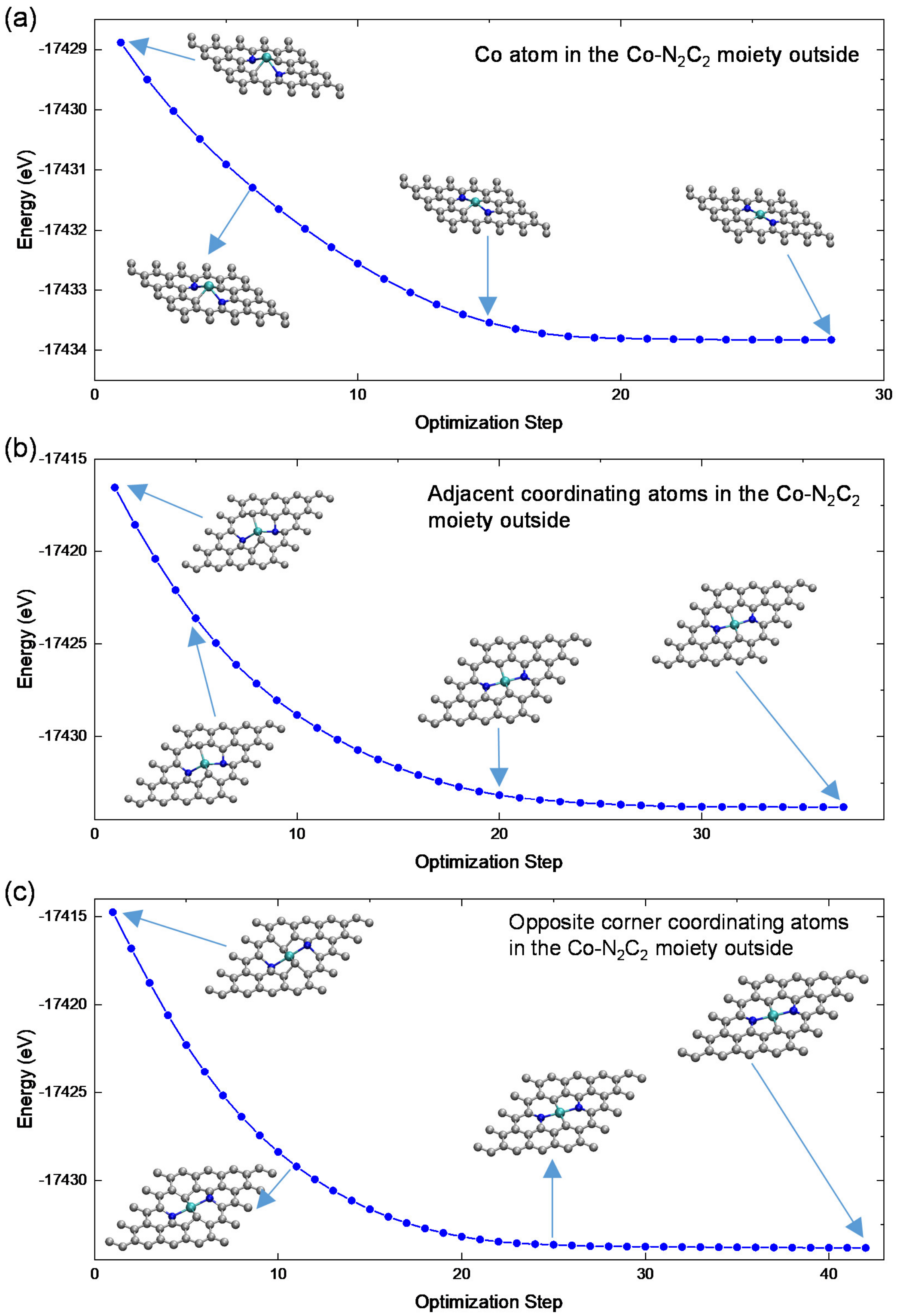

Supplementary Figure 8. Structural optimizations of Co-N_2C_2. Structural optimizations with non-planar Co-N_2C_2 moieties as initial structures.

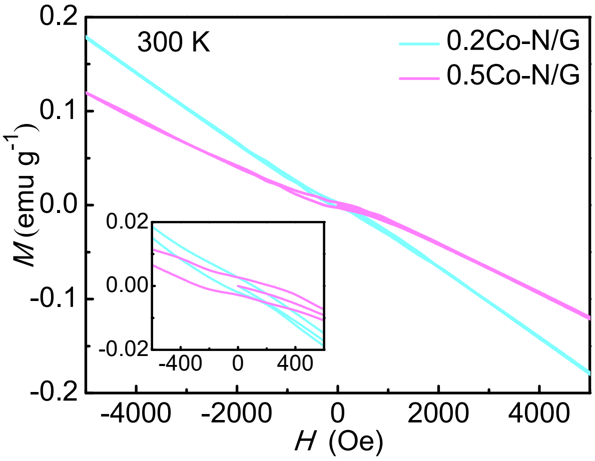

Supplementary Figure 9. Magnetic characterization. The raw data of *M*-*H* curves for 0.2Co-N/G and 0.5Co-N/G nanosheets at 300 K without background subtraction.

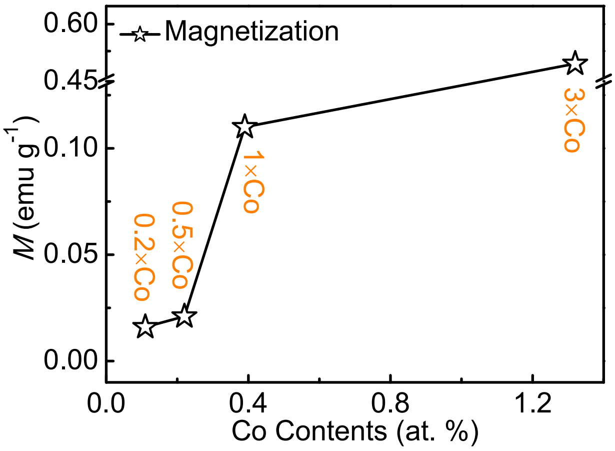

Supplementary Figure 10. Variation trend of magnetic moments.. The trend of macroscopic magnetic moments with variable Co doping contents at 300 K for 0.2Co-N/G, 0.5Co-N/G, 3Co-N/G and 1Co-N/G nanosheets.

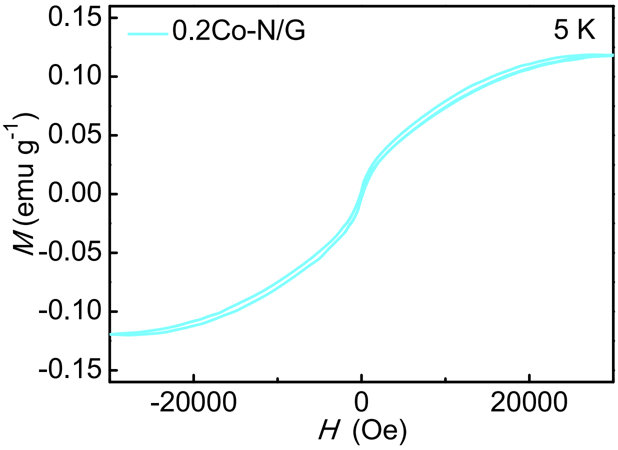

Supplementary Figure 11. Magnetic characterization. The *M*−*H* curve for 0.2Co-N/G nanosheets at 5 K after background subtraction.

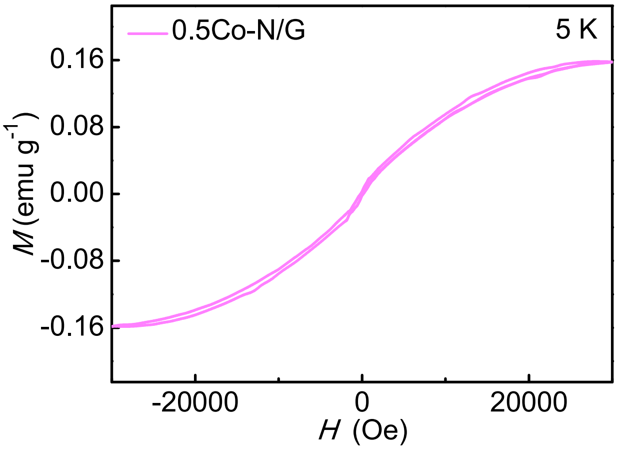

Supplementary Figure 12. Magnetic characterization. The *M*−*H* curve for 0.5Co-N/G nanosheets at 5 K after background subtraction.

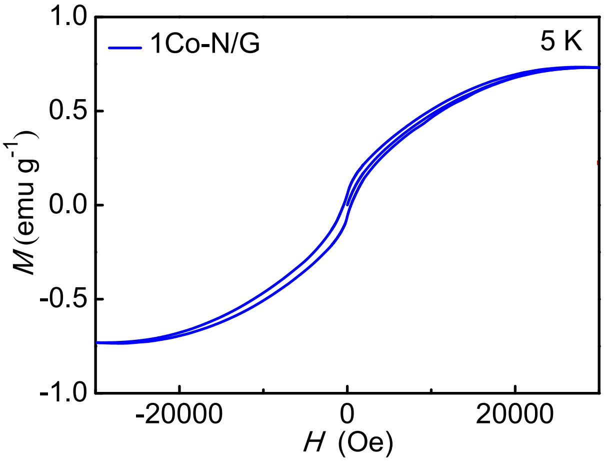

Supplementary Figure 13. Magnetic characterization. The *M*−*H* curve for 1Co-N/G nanosheets at 5 K after background subtraction.

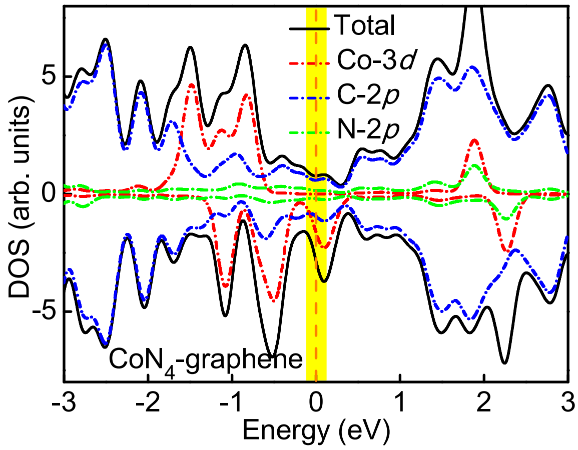

Supplementary Figure 14. DOS and PODS of CoN_4-graphene. The stack diagram of DFT calculated total and partial densities of states (DOSs) for CoN_4-graphene system.

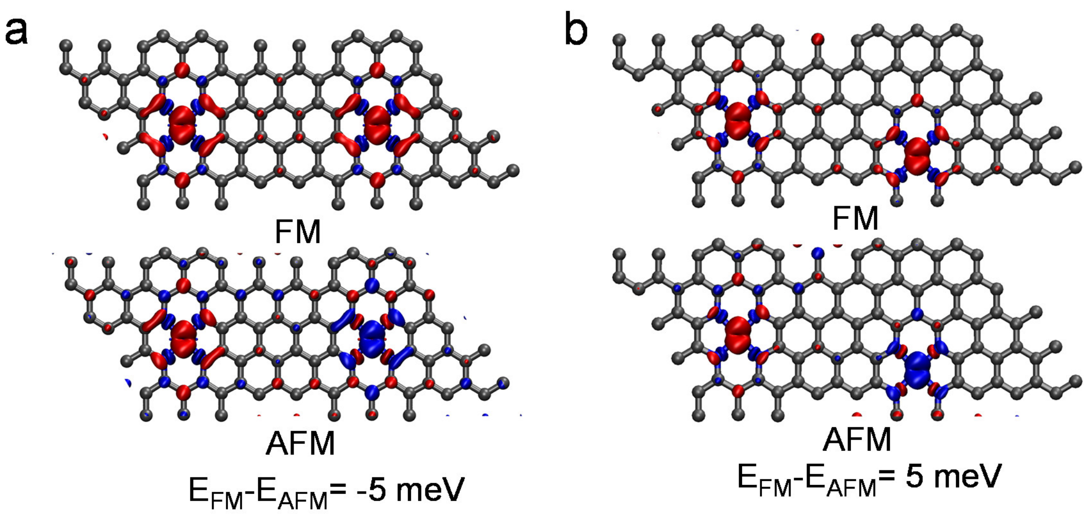

Supplementary Figure 15. The magnetic coupling of two Co-N_4 moieties embedded in graphene with different configurations. The spatial distribution of spin density for a 10×5×1 graphene supercell with two N_4-coordinated Co atoms, indicating that the coupling between two Co atoms can be either (a) FM or (b) AFM, depending on the relative positions in the context of the bipartite nature of graphene.

Supplementary Tables

Supplementary Table 1. The atomic contents of C, N and Co elements in 0.2Co-N/G, 0.5Co-N/G, 1Co-N/G and 3Co-N/G nanosheets.

Elements	C (at. %)	N (at. %)	Co (at. %)
0.2Co-N/G	85.33	7.83	0.11
0.5Co-N/G	87.13	7.96	0.22
1Co-N/G	86.21	7.24	0.40
3Co-N/G	84.8	8.15	1.32

Supplementary Table 2. Results of Co K-edge EXAFS fitting for 1Co-N/G nanosheets.

Path	*N*	*R* (Å)	σ^2 (×10^{-3} $Å^2$)	ΔE_0 (eV)	*R* (%)
Co-N^1	3.1	1.89	4.8	-4.9	0.12
Co-N^2	1.0	2.12	5.6		

N, coordination number; *R*, bonding distance; σ^2, Debye-Waller factor; ΔE_0, inner potential shift; *R* factor (%) indicates the goodness of the fit; S_0^2, the amplitude reduction factor, was fixed at 0.93 as determined by Co foil fitting.

References

1. Du Z*, et al.* Cobalt in nitrogen-doped graphene as single-atom catalyst for high-sulfur content lithium-sulfur batteries. *J. Am. Chem. Soc.* **141**, 3977-3985 (2019).
2. Zhu Y*, et al.* A cocoon silk chemistry strategy to ultrathin N-doped carbon nanosheet with metal single-site catalysts. *Nat. Commun.* **9**, 3861 (2018).
3. Jiang K*, et al.* Isolated Ni single atoms in graphene nanosheets for high-performance CO_2 reduction. *Energ. Environ. Sci.* **11**, 893-903 (2018).
4. Fei H*, et al.* Microwave-assisted rapid synthesis of graphene-supported single atomic metals. *Adv. Mater.* **30**, 1802146 (2018).
5. Zhao L*, et al.* Cascade anchoring strategy for general mass production of high-loading single-atomic metal-nitrogen catalysts. *Nat. Commun.* **10**, 1278 (2019).
6. Liang Y*, et al.* Covalent hybrid of spinel manganese-cobalt oxide and graphene as advanced oxygen reduction electrocatalysts. *J. Am. Chem. Soc.* **134**, 3517-3523 (2012).
7. Wang X*, et al.* Uncoordinated amine groups of metal-organic frameworks to anchor single Ru sites as chemoselective catalysts toward the hydrogenation of quinoline. *J. Am. Chem. Soc.* **139**, 9419-9422 (2017).
8. Wang C*, et al.* Atomic Fe embedded in carbon nanoshells–graphene nanomeshes with enhanced oxygen reduction reaction performance. *Chem. Mater.* **29**, 9915-9922 (2017).
9. Yuan K*, et al.* Synergetic contribution of boron and Fe–N_x species in porous carbons toward efficient electrocatalysts for oxygen reduction reaction. *Acs Energy Lett.* **3**, 252-260 (2018).
10. Zheng Y*, et al.* Hydrogen evolution by a metal-free electrocatalyst. *Nat. Commun.* **5**, 3783 (2014).
